# Tau interactome and RNA binding proteins in neurodegenerative diseases

**DOI:** 10.1186/s13024-022-00572-6

**Published:** 2022-10-17

**Authors:** Tomas Kavanagh, Aditi Halder, Eleanor Drummond

**Affiliations:** Brain and Mind Centre and School of Medical Sciences, Faculty of Medicine and Health, University of Sydney, 94 Mallett Street, Sydney, NSW Australia

**Keywords:** Tau, Interactome, Protein–protein interactions, Alzheimer’s disease, Frontotemporal dementia, Tauopathy, HNRNP, RNA binding proteins, Neurofibrillary tangle

## Abstract

Pathological tau aggregation is a primary neuropathological feature of many neurodegenerative diseases. Intriguingly, despite the common presence of tau aggregates in these diseases the affected brain regions, clinical symptoms, and morphology, conformation, and isoform ratio present in tau aggregates varies widely. The tau-mediated disease mechanisms that drive neurodegenerative disease are still unknown. Tau interactome studies are critically important for understanding tauopathy. They reveal the interacting partners that define disease pathways, and the tau interactions present in neuropathological aggregates provide potential insight into the cellular environment and protein interactions present during pathological tau aggregation. Here we provide a combined analysis of 12 tau interactome studies of human brain tissue, human cell culture models and rodent models of disease. Together, these studies identified 2084 proteins that interact with tau in human tissue and 1152 proteins that interact with tau in rodent models of disease. Our combined analysis of the tau interactome revealed consistent enrichment of interactions between tau and proteins involved in RNA binding, ribosome, and proteasome function. Comparison of human and rodent tau interactome studies revealed substantial differences between the two species. We also performed a second analysis to identify the tau interacting proteins that are enriched in neurons containing granulovacuolar degeneration or neurofibrillary tangle pathology. These results revealed a timed dysregulation of tau interactions as pathology develops. RNA binding proteins, particularly HNRNPs, emerged as early disease-associated tau interactors and therefore may have an important role in driving tau pathology.

**Supplementary Information:**

The online version contains supplementary material available at 10.1186/s13024-022-00572-6.

## Background

Tau is the central pathological protein in many types of neurodegenerative disease. Collectively, these diseases are referred to as “tauopathies” due to the key pathological role of tau. Tauopathies can be divided into primary tauopathies including corticobasal degeneration (CBD), Pick’s disease (PiD), progressive supranuclear palsy (PSP), argyrophilic grain disease (AGD), globular glial tauopathy (GGT), aging related tau astrogliopathy (ARTAG), primary age related tauopathy (PART), and chronic traumatic encephalopathy (CTE) or secondary tauopathies such as Alzheimer’s disease (AD). Despite the undisputed central role of tau in tauopathies, the mechanism(s) by which tau drives disease remains elusive. Additionally, it is unknown if tau drives disease via the same mechanism across all tauopathies. These critical knowledge gaps are partly due to the complexity of tau: there are 6 isoforms of tau in the human brain, all of which differently accumulate in tauopathies (e.g. aggregates of 3R tau isoforms accumulate in Pick’s disease, aggregates of 4R tau isoforms accumulate in PSP and CBD [37]. Furthermore, tau is the most post-translationally modified protein in neurodegenerative diseases. 118 post-translational modifications have been observed on tau in human AD brain tissue, 68 in CBD, 44 in GGT, 33 in PSP, 46 in PiD and 5 in AGD. This is very important from a disease mechanism perspective as many of these post-translational modifications differently influence the aggregation dynamics, toxicity, and protein–protein interactions of tau [46, 113]. In addition, the conformation of tau is different in each tauopathy, which further influences protein interactions and function [69]. Despite these challenges, recent studies have made significant progress in uncovering tau-mediated disease mechanisms, particularly by studying tau protein–protein interactions. Intriguingly, many of these studies have consistently observed pathological interactions between tau and RNA binding proteins in tauopathies. Here, we provide a comprehensive review of tau protein–protein interactions in tauopathies and highlight how the interaction between RNA binding proteins and tau may mediate disease in tauopathies.

## The protein tau

The specific type of tau species is an important determinant of protein interactions and downstream pathological consequences. The *MAPT* gene (microtubule associated protein tau) encodes six protein isoforms commonly expressed in adult neurons [7, 104]. These vary structurally by the inclusion of 0, 1 or 2 N-terminal repeats (denoted as 0 N, 1 N and 2 N, exons 2 and 3) and the inclusion of three or four microtubule binding domains (denoted as 3R or 4R, exon 10) [7, 104]. In healthy neurons there is an equivalent amount of 3R and 4R tau. These isoforms have distinct but overlapping functions and protein–protein interactions [71]. Interestingly, central nervous system neurons extending into the periphery and peripheral neurons express a high molecular weight isoform of tau whose function remains unknown [34, 40]. This larger form of tau arises from the inclusion of a large exon (exon 4a) between the N-terminal repeats and proline rich region, and may limit tau aggregation propensity in the peripheral nervous system. Tau isoforms are regulated by a wide array of RNA-binding proteins and splicing factors (including HNRNPA1, HNRNPE2/E3, HNRNPG, HNRNPK, SRSF2, and PTB [20, 72, 111]). Tau is expressed robustly throughout the human brain with isoform specific expression at different development stages, different regions, and in select cell populations [17, 22, 73, 81, 104].

Understanding the different roles tau isoforms can play in a cell is critical to deciphering disease processes. For example, 4R isoforms show the greatest propensity to aggregate and lead to cognitive defects in animal models [95, 97], while, 0N3R tau isoforms lead to axonal transport defects and reduce the life-span of *drosophila* models [97]. Tau isoforms are differentially distributed within neurons, with 2 N tau isoforms showing enrichment in neuronal cell bodies whilst 0 N and 1 N tau isoforms are sorted to axons [13, 70] where they alter microtubule dynamics [90]. 1 N tau isoforms have also been found in the nucleus [70, 75] where tau has been reported to mediate ribosomal gene expression, which has been implicated in proteostasis disruption in tauopathy [31], and epigenetic and heterochromatin changes especially during stress events [49, 62, 76, 106]. Furthermore, tau isoforoms may interact differently with disease associated proteins such as APOE [71], which may lead to disease specific interactions where one tau isoform is altered.

Importantly tau also changes its localisation in neurons post injury or in disease states. Canoncially tau is believed to be enriched in axons of neurons but redistributes to the somato-dendritic compartment or dendritic spines when exposed to trauma, amyloid-beta, hyperphosphorylation by FYN kinase and other signals. It has also been shown that the mutant P301L tau mislocalises to dendritic spines in FTLD [53]. However, a recent study has cast doubt on whether tau mislocalises to the somato-dendritic compartments or is already abundant in this region prior to disease [91]. These disparities are possibly due to the difficulty in detecting tau isoforms or inappropriate localisation of tau in model systems. Regardless, it is important to consider the localisation of tau in the context of interactions as mislocalisation of tau will influence its interaction profile.

## Tau isoforms are dysregulated in tauopathies

In physiological conditions, tau is a soluble and unstructured protein. However, in tauopathy it oligomerises and then forms aggregates with a range of conformations [38], resulting in distinct neuropathological lesions in each tauopathy [26]. Many tauopathies have a bias towards one tau isoform over the others [21, 38, 87, 103] and the isoform of tau appears to influence the rate of aggregation with 4R isoforms aggregating the most rapidly [121]. It is not yet understood why this is the case. For example, PiD displays increased aggregation of 3R tau [33] as compared AGD, CBD, GGT and PSP which all have a bias for developing 4R tau aggregates [26, 37, 103]. Neurofibrillary tangles in AD contain both 3R and 4R tau but the ratio of 3R:4R tau in tangles shifts based on the maturity and location of the aggregate [23].

## Post-translational modifications of tau in tauopathies

**Fig. 1:**
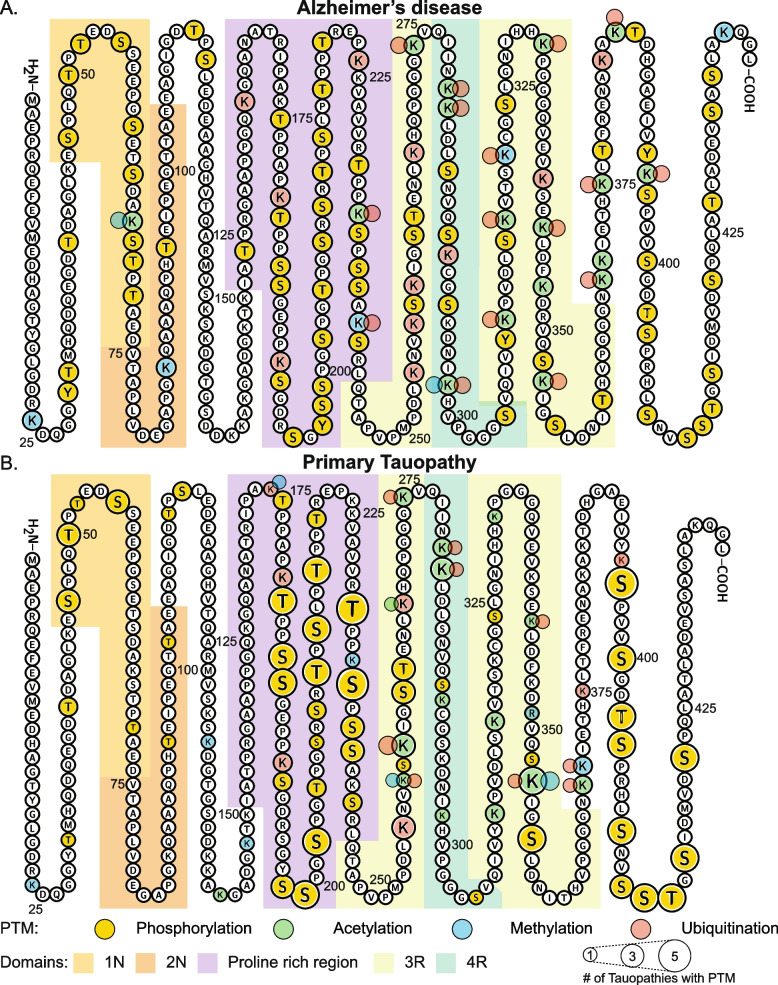
Summary of PTMs present on tau in AD and primary tauopathies. **A** PTMs observed in AD mapped to full length tau and **B** in primary tauopathies; AGD, CBD, GGT, PiD, PSP. Alternatively spliced regions are coded light orange = 1 N, dark orange = 2 N and light green = 3R, dark green 4R and purple for the proline rich region. Compiled from [1, 10, 29, 30, 36, 50, 58, 78, 92, 94, 113, 115]. Please note that while this compilation of tau PTMs was completed to the best of our knowledge, this is an evolving field and may not be comprehensive

Post-translational modifications (PTMs) of tau also significantly influence protein interactions, sub-cellular localisation, and aggregation propensity of tau [21, 66, 74]. Tau undergoes a vast range of PTMs including phosphorylation, ubiquitination, acetylation, methylation, glycosylation, glycation, oxidation and SUMOylation (for review see [5]). Identification of specific PTM sites involved in human disease historically occurred through antibody-based techniques; however, this approach is not a comprehensive method of identifying all PTMs. The use of mass spectrometry has allowed a more comprehensive analysis of PTMs of tau in post-mortem human tauopathy brain tissue, overcoming these limitations of disease models and antibody-based approaches. Recent mass spectrometry studies have identified numerous additional phosphorylation sites on tau, and further characterised tau’s less abundant PTMs including acetylation, methylation, and ubiquitination [58, 113]. Most studies analysing PTM sites present in human tauopathy brain tissue have focused on AD tissue, with a small number of studies examining CBD, PSP and Pick’s disease tissue [1, 10, 29, 30, 47, 50, 58, 78, 92, 115]. Limited studies have examined PTMs directly in post-mortem tissue in GGT and AGD [47, 58, 94], and no studies to date have analysed tau PTMs in PART. A summary of tau PTMs present in human brain tissue across a range of tauopathies is presented in Fig. 1 and Supplementary Table 1.

Patterns of phosphorylation differ across tauopathies, with AD, CBD and PiD most frequently phosphorylated, followed by GGT and PSP (Fig. 1; Supplementary Table 1). Few phosphorylation sites have been identified in AGD (Fig. 1). The most frequent phosphorylation sites across these diseases cluster within the proline-rich region, with an additional common cluster in the C-terminal domain (Fig. 1). In contrast, acetylation predominantly occurs upon lysine residues within the microtubule binding regions. These same residues can also be involved in ubiquitination and methylation. Both AD and GGT have multiple lysine residues (K274, K280, K281) where acetylation and ubiquitination compete for common sites. Ubiquitination of tau is a feature of all tauopathies. Interestingly, ubiquitination in the C-terminal domain appears unique to AD (Fig. 1) and models show this PTM stabilizes binding between protofilaments within AD straight filaments [10]. Similarly, cryo-EM suggests K353 ubiquitination in CBD may stabilise packing between protofilaments, with competing acetylation at this site changing filament structure and interactions between filaments [10]. Further combined cryo-EM and mass spectrometry studies of different tauopathies could elucidate the role of specific PTMs in mediating tau fibrillization and aggregation in human disease.

Patterns of PTMs unique to certain tauopathies have previously been identified in the literature, such as the absence of phosphorylation at S262 in PiD, in contrast to its presence in AD, CBD, GGT and PSP [58]. This may relate to structural differences unique to tau filaments in PiD, a 3R tauopathy, with S262 falling within the core region in PiD instead of its location outside the core in AD and other tauopathies (Supplementary Table 1). In conjunction with a tight turn in PiD tau filaments at G261, it is hypothesised that the core location of S262 in PiD may reduce its accessibility to kinases [33]. However, the mechanisms behind other absent PTMs in PiD, such as T263 and S412 phosphorylation, and K281 acetylation, are yet to be explored, or their relevance in human disease clarified. Similarly, PSP’s PTM signature includes an absence of T50, S237 and S238 phosphorylation, contrasting AD, CBD, GGT and PiD (Supplementary Table 1). With a lack of specific biomarkers for sporadic forms of FTD [100], the identification of these nuances in PTMs between AD, CBD, GGT, PSP and PiD could support investigation of new biomarker assays that could diagnose non-AD tauopathies.

Temporal variations in PTMs also exist within tauopathies, with the phosphorylation pattern of tau sequentially changing during AD, with T231 increased significantly by Braak stage III/IV, but other residues (such as S199, S202/T205, S422) significantly increased only at Braak stage V/VI in human AD brains [89]. S396 phosphorylation was present in all tauopathies involving analysis of human post-mortem tissue (Supplementary Table 1). While there is a general perception in the field that phosphorylation of S396/S404 (identified using the PHF1 antibody) occurs later in the disease process, multiple recent studies show that phosphorylation of S396 is actually observed very early in disease in both PSP and AD, suggesting that it may be a feature of early stage disease [9, 78]. *In vitro* studies in HEK-293 cells suggest kinases involved in AD such as GSK3β require stepwise tau phosphorylation before S396 phosphorylation, with S404 and S400 requiring phosphorylation beforehand [68]. However, the impact of these different spatial and temporal patterns of PTMs of tau, and relevance to human disease remain under investigation. The identification of sequential patterns of PTMs in tauopathies holds the potential to help distinguish early from late-stage disease.

## The unique conformations of tau aggregates in tauopathies

Tau undergoes multiple stages of aggregation during tauopathy progression from monomeric soluble tau to small oligomers to large aggregates. These changes complicate the understanding of how tau conveys toxicity as these different aggregate conformations likely have different interactomes. Oligomers are present earlier in disease progression, have higher propensity for seeding, and their small size allows greater mobility and potential to disrupt physiological tau interactions [42, 56, 64]. Oligomers are considered more toxic than large insoluble aggregates [11] as they generate more pathology than fibrils when seeded into mice [56]. Furthermore, Ghag et al. [42] convincingly demonstrated that tau oligomers are the toxic species *in vitro*, while tau fibrils are comparatively inert.

Recent cryo-EM studies have confirmed that the conformation of tau is different between tauopathies. For example, tau aggregates into straight or paired helical filaments in AD [3, 35]. In PiD, aggregates have a sharp folded beta-sheet that can align end-to-end to form a wide filament from two narrow filaments [33, 96]. Tau aggregates in CBD have a non-proteinaceous molecule in the centre of the folded structure [119]. Importantly, recombinant tau aggregates have a different conformation than aggregates extracted directly from human diseased brain tissue, highlighting a significant caveat associated with using recombinant tau in experimental studies [118]. Tau aggregates purified from AD, PiD, PSP and CBD post-mortem brain tissue replicate disease-specific pathology in both tissue culture and mouse models of disease, suggesting the disease-specific tau aggregates characterise each tauopathy [2, 19, 25, 27, 28, 51, 64, 88, 114]. Pathological aggregate ‘strains’ maintain their preference for cell types and regional specificity when injected into mouse brains [19, 51, 88], suggesting that the microenvironment and specific interactions of tau are important factors in driving pathology. Furthermore, whilst 3R or 4R aggregates can seed aggregates primarily composed of either isoform, they are much more efficient at seeding the aggregation of conformers of the same isoform [1, 51, 101, 117]. Together, the differences in regional vulnerability, preference for tau isoforms and distinct aggregate structures that are unique in each tauopathy suggest that different underlying mechanisms may drive disease in each tauopathy.

## Tau interactome studies reveal consistent interaction with RNA binding proteins

Analysis of the tau interactome, particularly the analysis of the interactome of pathological tau strains, is an excellent way to define disease mechanisms that drive tauopathies. The protein interactions of tau are influenced by several factors such as cellular location, isoform, aggregation state, conformation, and the presence of PTMs. Therefore, analysis of strain- and disease-specific tau interactomes provides a unique opportunity to uncover disease mechanisms that drive tauopathies. While research in this area is still in its infancy, several studies have investigated the tau interactome in a variety of models and post-mortem human brain tissue. These studies have primarily used affinity purification-mass spectrometry or localised proteomics to examine tau interactions. Most of these studies have either analysed the total tau interactome or have limited their analysis to full length (2N4R) tau. Furthermore, tau interactome studies have been largely carried out in the context of AD (balanced 3R/4R tau or AD post-mortem human tissue) or examining the interactome of tau expressing mutations present in rare familial cases of FTLD (P301L or V337M) (summarised in Table 1).

**Table 1:** Summary of tau interactome studies and localized proteomics studies of tau aggregates

Study	Species	Model	TYPE OF STUDY	Tau Target	Replicates
**Ayyadevara et al., 2016 **[12]	Human	Post-mortem brain tissue	Interactome	Total tau (tau-5)	4 AD, 4 Control
**Drummond et al., 2020 **[30]	Human	Post-mortem brain tissue	Interactome	PHF1 tau	5 AD
**Hsieh et al., 2019 **[54]	Human	Post-mortem brain tissue	Interactome	Total tau (tau-5)	4 AD, 4 Control
**Meier et al., 2015 **[82]	Human	Post-mortem brain tissue	Interactome	Total tau (tau-5)	3 AD, 3 Control
**Gunawardana et al., 2015 **[48]	Human	SHSY5Y cells	Interactome	Full length tagged tau	3 replicates
**Tracy et al., 2022 **[105]	Human	Human IPSC derived excitatory neurons	Interactome	Full length tagged tau and (WT, V337M and P301L)	7 replicates WT tau, 8 replicates V337M and P301L tau
**Wang et al., 2019 **[110]	Human	IMR-31 and ReN VM cells	Interactome	3R, 4R or 4R^P301L^ GFP tau constructs	3 replicates
**Jiang et al., 2021 **[57]	Mouse	C5Bl6/J Primary Neurons	Interactome	4R1N-tau:mCherry:Cry2Olig construct	3 replicates 4R1N-tau:mCherry:Cry2Olig construct, 3 replicates mCherry:Cry2Olig construct
**Liu et al., 2016 **[71]	Mouse	C5Bl6/J and Tau KO	Interactome	Total tau, 0 N, 1 N and 2 N tau	3 total tau, 1 each of 0 N, 1 N and 2 N tau
**Maziuk et al., 2018 **[79]	Mouse	rTg4510, PS19 and TIA1-/- C5Bl6/J	Interactome	Total tau (tau-13)	4 rtg4510, 4 C57BL6/J
**Sinsky et al., 2020 **[99]	Rat	Humanised tau	Interactome	Total tau (DC18, DC190 and DC25 tau)	2 transgenic, 2 control
**Wang et al., 2017 **[109]	Mouse	PS19	Interactome	Total tau (tau-5, tau-21, tau-24)	4 replicates
**Hondius et al., 2021 **[52]	Human	Post-mortem brain tissue	Localized proteomics	GVD and NFT positive neurons	12 AD, 12 Control, 3000 neurons per sample
**Drummond et al., 2020 **[30]	Human	Post-mortem brain tissue	Localized proteomics	NFT pathology	7 AD, 4000 NFTs per sample
**Wang et al., 2005** [112]	Human	Post-mortem brain tissue	Localized proteomics	NFT pathology	4 AD, ~ 2000 total NFTs

Here, we have performed a comprehensive review followed by a combined analysis of tau interactome studies to identify and compare tau interactors in human post-mortem tissue, human cell culture models, and rodent models. Literature review identified twelve tau interactome studies that satisfied our search criteria and provided sufficient data for re-analysis (Table 1). To allow for direct comparison between studies we used a vote-counting approach and gene ID as the protein identifier. Tau interactors were filtered to those that appeared in at least 3/7 human studies and 2/5 rodent studies.

Four studies analysed tau interactions in AD post-mortem human brain tissue. Two assessed the interactome of total tau in AD brain homogenates vs cognitively normal age-matched controls [12, 54], one assessed the interactome of total tau in endoplasmic reticulum enriched fractions of AD vs cognitively normal age-matched controls [82], and one assessed the PHF-1 immunoreactive phosphorylated tau interactome in AD brain tissue [30]. 3 additional studies analysed the tau interactome in human cell models; Gunawardana et al. [48] analysed the tau interactome in SH-SY5Y neuroblastoma cells transfected with GFP tagged 2N4R tau (with or without P301L mutation), Wang et al. [110] analyzed the tau interactome in CRISPR engineered human neural progenitor ReNCell VM cells and Human Neuroblastoma IMR-32 cells expressing equal amounts of GFP tagged 3R and 4R tau, and Tracy et al. [105] analyzed the tau interactome in human induced pluripotent stem cells (IPSCs) expressing APEX-fused 2N4R tau (with and without either P301L or V337M mutations). Importantly, the interactomes of these tagged tau constructs appear broadly consistent with other studies.

**Fig. 2:**
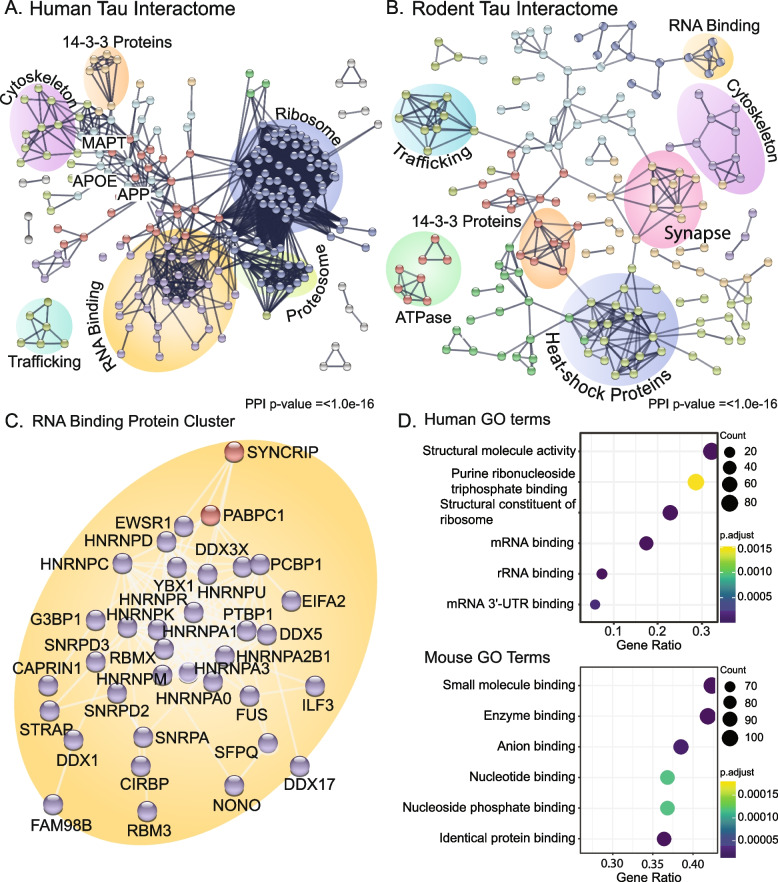
Tau interactome analyses reveal consistent tau-protein interactions across human brain and cell culture models. **A** PPI network of human tau interacting proteins consistent in > 3 of 7 studies. Network node colours are clustered by k-means set with 8 groups. String network is limited to physical interactions only. Clusters of interacting proteins are manually annotated (see Supplementary Table 2 for annotated proteins). **B** PPI network of rodent model tau interacting proteins. Network node colours are clustered by k-means set with 8 groups. String network is limited to physical interactions only. Clusters of interacting proteins are manually annotated (see Supplementary Table 3 for annotated proteins). **C** Zoomed in view of PPI network of RNA-binding proteins in human tissue **D** GO Molecular function enrichments of human and rodent models generated in R with the package clusterProfiler v4.2.2. Top 8 processes are listed. The background gene set for rodent and human studies included all proteins detected in IP studies (see Supplementary Table 4 for GO molecular function annotations). All PPI networks were generated with the STRING database (v11.5, https://string-db.org/)

Combined analysis of the 7 studies assessing tau interactions in human post-mortem tissue or human cell lines identified 2084 tau interacting proteins (Supplementary Table 2). Of these, 261 proteins were consistently found in > 3 studies. 72 of these proteins interacted with both phosphorylated tau and total tau, and 253 proteins also interacted with either P301L or V337M mutant tau (Supplementary Table 2). Protein–protein interaction network analysis of the 261 proteins that interacted with tau consistently across > 3 studies showed particularly significant enrichment of RNA binding proteins and ribosomal proteins (Fig. 2.A). The most consistent tau-interacting RNA binding proteins were heterogeneous nuclear ribonucleoproteins (HNRNPs), FUS, SFPQ and PTBP1 (Table 2, Supplementary Table 2). The consistent interaction between tau and RNA binding proteins across multiple studies is particularly interesting as RNA is a known component of tau aggregates in tauopathies [44, 45] and RNA (especially tRNAs) enhances the aggregation of tau [59, 120]. Furthermore, previous studies suggest that RNA can be required as an intermediate linker for select tau-protein interactions, particularly with ribosomal proteins [48]. However, it is important to note that this is not always the case; for example, several HNRNP-tau interactions are enhanced after RNA removal [48].

**Table 2:** Summary of tau interacting HNRNPs and select disease associated RNA binding proteins from human studies

RBP	Number of Studies	pTau Interactor	APEX tagged	Interacts with Mutant Tau	Present in NFT	Altered in GVD Neurons	Altered in NFT Neurons	Interacts with Mutant Tau
*HNRNPK*	4	Yes	No	Yes	Yes	No (Increased)	No	Yes
*HNRNPA1*	4	Yes	Yes	Yes	Yes	No (Increased)	No	Yes
*HNRNPA2B1*	4	Yes	No	Yes	Yes	No (Increased)	No	Yes
*HNRNPA3*	4	No	No	Yes	Yes	No	No (Decreased)	Yes
*HNRNPA0*	3	No	No	Yes	No	No	No (Decreased)	Yes
*HNRNPC*	3	No	No	Yes	Yes	No	No	Yes
*HNRNPD*	3	No	No	Yes	Yes	No	Yes (Decreased)	Yes
*HNRNPM*	3	No	No	Yes	Yes	No (Decreased)	Yes (Decreased)	Yes
*HNRNPR*	3	No	No	Yes	Yes	No	Yes (Decreased)	Yes
*HNRNPU*	3	No	No	Yes	Yes	Yes (Decreased)	Yes (Decreased)	Yes
*PTBP1*	4	No	No	Yes	No	No	No	Yes
*FUS*	3	No	No	Yes	Yes	No	Yes (Decreased)	Yes
*SFPQ*	3	No	No	Yes	Yes	No	No (Increased)	Yes
*G3BP1*	3	No	No	Yes	No	No	NA	Yes
*NONO*	3	No	No	Yes	Yes	No	NA	Yes
*PABPC1*	3	No	Yes	Yes	Yes	No	NA	Yes
*TARDBP*	1	No	No	Yes	Yes	Yes (Decreased)	Yes (Decreased)	Yes
*TIA1*	N.D	N.D	N.D	N.D	N.D	N.D	N.D	N.D

Table shows the number of human tau interactome studies each RNA binding protein (RBP) has been identified in, if there is an interaction between the RBP and phosphorylated tau (pTau; [30]), if the RBP interacts with tau in a human cell culture model using APEX [105], if it is present in neurofibrillary tangles (NFTs; [30], if protein levels are altered in neurons containing granulovacuolar degeneration or NFTs (Yes/No indicates whether it passed FDR < 0.05 whilst ‘Increased/Decreased’ denote a greater than 10% change in estimated protein expression, N.D. denotes protein not detected; [52]).

Comparison of the P301L mutant and wild-type tau interactomes highlighted reduced interaction between tau and proteasome subunits and increased interaction between tau and 40/60S ribosomal subunits, translation initiation factors and HNRNPs [48]. This provides key evidence that different tau strains have different interactomes, which provides downstream avenues for future research investigating disease mechanisms. Two of the most consistent clusters of proteins in the human tau interactome are proteosome and ribosome proteins (Fig. 2.A, D). The proteosome is frequently dysregulated in tauopathy. Tau aggregates or tau overexpression lead to altered proteostasis and protease inhibition [18, 116] and small molecules activating the proteosome can reverse cognitive deficits associated with tau [86]. Almost all consistent tau interacting proteosome proteins also interact with phosphorylated tau, suggesting that these interactions may be enhanced at later stage pathology [60] and may reflect disease-associated tau inhibiting the proteosome in a manner similar to amyloid-beta and α-synuclein [80, 102]. The interaction between tau and the ribosome has been a growing area of interest in the study of tauopathy. Tau interacts with, and sequesters, ribosomal components – particularly regulatory components [32, 63], resulting in decreased protein synthesis in models of tauopathy [31]. Ash et al. [11] demonstrated that TIA1 can readily drive the aggregation of tau alongside other RNA binding proteins which required crowding agents to drive aggregate tau. Furthermore, tRNAs increase the rate of tau aggregation in vitro [120], and as such the ribosome may act as a seeding point for tau oligomerisation and aggregation. Interestingly ribosome proteins don’t appear robustly enriched in interactions with phosphorylated tau, suggesting tau may play a consistent role in ribosomal regulation that is altered with disease state and not required for late-stage pathology. Whether the interaction between tau and ribosomal proteins interrupts specific populations of translating ribosomes is yet to be fully explored.

Five studies have analysed the tau interactome in rodents. Liu et al. [70] assayed total tau, 3R tau, and 4R tau specific interactions in mice. Wang et al. [109] investigated full-length tau interactions of PS19 mice (expressing P301S mutant tau). Maziuk et al. [79] investigated total tau interactomes in wild type and rTg4510 mice (expressing P301L human mutant tau) and Sinsky et al. [99] explored the interactomes of 2N4R tau in wild type and transgenic humanised tau rats. Jiang et al. [57] assessed the interactions of inducible oligomerising tau constructs in mouse primary neurons. These rodent studies detected fewer total proteins than the human studies described above: with 244 of 1,152 (15.9%) detected proteins present in 2 or more rodent tau interactome studies (Supplementary Table 3). This inconsistency and lack of detection is likely a result of the wide variety of tau species studied, the low numbers of animals included in each study, and the fact that humanised mutant tau may not interact well with mouse RNA binding proteins and ribosomal proteins. Importantly, many of the consistent protein interactions with tau that were observed in human studies were not observed in rodent studies (Fig. 2.B). For example, while some studies did report interactions between tau and some HNRNP proteins (for example Hnrnpa2/b1 and a3 in rats [99] and Hnrnpa0, l, k, u, r and q in rTg4510 mice [79] and Hnrnpa0, a1 and a2b1 in inducible oligomeric tau expressing C7Bl6/J primary neurons), interactions between tau and RNA binding proteins was not a consistent feature of rodent studies. Additionally, interactions between tau and ribosomal proteins were not consistently observed in rodent studies (Fig. 2.B). Rodent studies showed consistent enrichment of tau interactions with heat shock proteins, and energy metabolism, synapse, and trafficking proteins (Fig. 2.B, D). Despite the inconsistencies noted between human and rodent studies of the tau interactome, some interactions were maintained between species. For example, interaction between tau and proteins involved in vesicle trafficking and the unfolded protein response were observed in both humans and rodents. This highlights some important caveats to consider when using rodent models of disease to study disease mechanisms present in human tauopathies.

## Association of tau interacting proteins with pathology

One important unanswered question is whether protein interactions with tau influence the development or progression of tau pathology in tauopathies. Two recent studies have used localized proteomics to analyze the proteins present in neurons containing neurofibrillary tangles (NFTs) or granulovacuolar degeneration (GVD; pathology hypothesized to be the precursor to the development of NFTs) isolated from AD human brain tissue [30, 52]. Integration of these two studies with our combined analysis of the human tau interactome identified proteins that are both present in pathological aggregates and interact with tau, and are therefore more likely to have a mechanistic role in the development of tau pathology in AD.

**Fig. 3:**
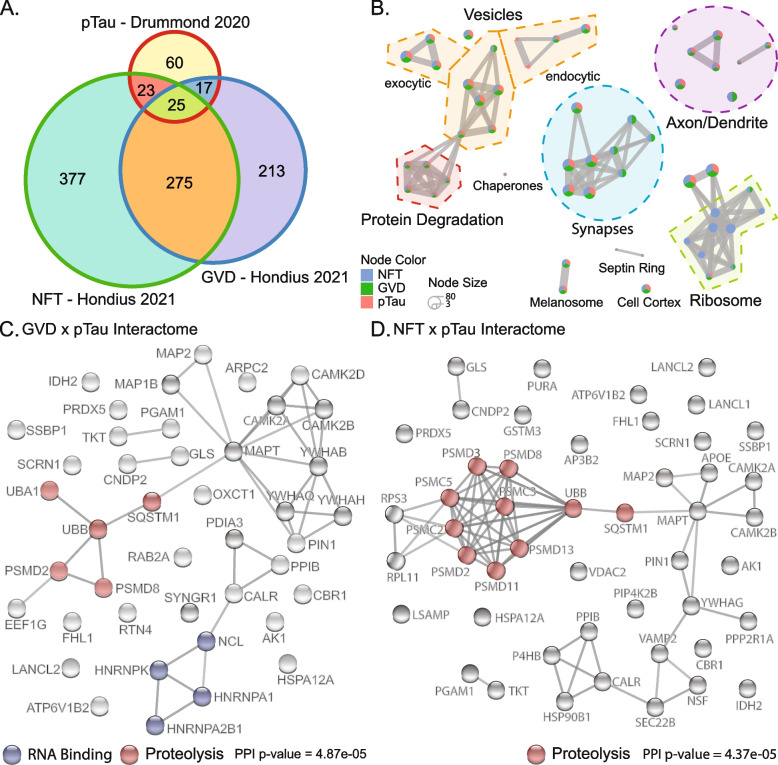
Analysis of proteins that interact with pTau and are altered in NFT or GVD containing neurons. **A** Venn diagram showing the overlap between pTau interacting proteins of Drummond et al. [30] and proteins with a trended change in expression in NFT or GVD containing neurons [52]. This trend was defined as a fold change in expression > 0.1 either up or down as p-values were not supplied with this dataset only FDR and most other proteomics studies fail to report FDR. **B** Enrichment plot summarising GO cellular compartment terms enriched for each dataset generated in R with clsuterProfiler v4.2.2. Nodes are divided into pie charts that show the proportion of genes shared for that category. Nodes are connected by shared genes to cluster terms into broader functions. Only the top 50 terms are shown. Clusters are annotated based on the majority of component pathways (Supplementary Table 6). **C** PPI network of proteins that are pTau interactors and altered in GVD and **D** NFT containing neurons. PPI networks were generated with the STRING database (v11.5, https://string-db.org/). String nodes are coloured by selected STRING annotation for those proteins (RNA binding and proteolysis). STRING network included physical and regulatory interactions

165 of the 261 consistent tau interactors were present in NFTs [30], showing that many tau interacting proteins were associated with pathological aggregates in AD. 37 tau interacting proteins were significantly altered in GVD containing neurons and 64 tau interacting proteins were significantly altered in NFT containing neurons [52]. We then specifically analysed which phosphorylated tau interacting proteins were enriched in neurons containing GVD or NFTs. This analysis showed that neurons containing GVD were significantly enriched in phosphorylated tau interacting proteins (42 proteins; p = 1.64*10^–5^, Fisher exact test, Fig. 3.A, Supplementary Table 5), as were neurons containing NFTs (48 proteins; p = 1.63*10^–4^, Fisher exact test, Fig. 3.A, Supplementary Table 5). These phosphorylated tau interacting proteins that were enriched in GVDs or NFTs included many with functions associated with the ribosome, synapses, and vesicle trafficking (Fig. 3.B, Supplementary Table 6). Tau interacting proteins altered in neurons containing NFTs largely included proteins involved in protein degradation pathways (Fig. 3.B, C, D, Supplementary Table 6). Given the consistent interaction between tau and RNA binding proteins described above, we were particularly interested to analyse whether altered levels of RNA binding proteins were associated with tau pathology. The majority of RBPs showed a trend for lower levels in GVD and NFT containing neurons (Supplementary Table 2). Noteworthy exceptions included HNRNPK, HNRNPA1 and HNRNPA2/B1, which all showed a trend for increased expression in GVD containing neurons (Fig. 3.C). Interestingly, these three HNRNPs all interacted with phosphorylated tau and their trend for increased expression was only observed in neurons containing early-stage pathology; increased expression was no longer observed in neurons containing NFTs. The interaction between total tau and these three HNRNPs is highly consistent in human studies including both in cell models and post-mortem brain tissue (Table 2). This suggests that not all HNRNPs have the same actions in tauopathy and lends weight to the hypothesis that HNRNPK, HNRNPA1 and HNRNPA2/B1 may influence the development of early tau pathology. The differential enrichment of these HNRNPs in neurons with GVD pathology and NFTs suggests there may be multiple phases to the dysregulation of tau interactions in disease with early stress response proteins present in GVD.

## HNRNPs: general biology and dysregulation in tauopathy

Our comprehensive review of tau interactome studies suggest that the interaction between HNRNPs (and more broadly RNA binding proteins) and tau may have an important role in tauopathy disease mechanisms. HNRNPs have broad molecular functions including splicing regulation, mRNA transport and stability, and mediating mRNA stress responses via stress granule formation [15, 41]. Their activity and binding are targeted to sub-classes of mRNA (e.g. binding m^6^A mRNAs by HNRNPA2/B1 [4, 57]). Understanding the interaction of HNRNPs with tau in disease contexts is critical as these proteins have the immediate ability to impact multiple specific pathways within a cell if dysregulated. Mutations in HNRNPs are associated risk variants for neurodevelopmental and neurodegenerative disorders (including ALS/FTLD, multisystem proteinopathy, Bain-type and HNRNPR-related or HNRNPU-related disorders amongst others [43, 61]). Furthermore, HNRNPs readily aggregate in disease and can enhance the rate of conformational change for other disease associated proteins such as TDP43 [16, 24, 77, 93]. Importantly, reduction of RNA binding proteins TIA1 and HNRNPA2/B1 expression has been shown to reduce or prevent the formation of tau pathology [57, 107].

While there is consistent evidence of tau interacting with HNRNPs (and with RBPs more broadly), the consequences of these interactions in disease contexts are largely unexplored. One hypothesis is that tau binds to RBPs, the ribosome and RNAs as part of the stress granule response. TIA1 – an RNA binding protein – has been shown to regulate the toxicity and aggregation of tau. TIA1 exacerbates tau aggregation with RNA and drives the generation of toxic tau oligomers *in vitro* and in cell models and reduction of TIA1 is protective against tau toxicity [11, 107, 108]. In addition, tau accelerates the formation of TIA1 positive stress granules [107]. *In vivo*, TIA1 regulates the toxicity of tau oligomers. Reduction of TIA1 inhibits the spread and prevents toxicity of seeded tau aggregates, but interestingly, increased neurofibrillary tangle burden and neuroinflammation [8, 56, 65]. TIA1 and other RNA binding proteins co-aggregate with tau in small lesions in rTg4510 mice and AD post-mortem brain tissue but appear adjacent to (not in) larger aggregates [56, 79]. While this interaction is increased with pathology, it is important to note that tau also interacts with RBPs and the ribosome in cell models without significant pathology or stress [48, 105, 110], suggesting these interactions occur in physiological conditions but are increased and stabilised in pathological conditions which then leads to detrimental effects for the cell. However, none of the affinity purification-mass spectrometry studies above detected TIA1 as a tau interactor. The interaction between TIA1 and tau has only been observed in rTg4510 mice with moderate/severe phenotypes or mammalian cells overexpressing both TIA1 and 0N4R tau, suggesting that the interaction between TIA1 and tau may be either of low abundance or indirect.

Oligomeric tau has been shown to co-aggregate with Musashi 1 and 2 in inducible HEK-293 cells [85] and with tau aggregates in AD and FTD [84, 98]. These aggregates develop in the cytoplasm and nucleus, impede Musahsi 1/2 nuclear/cytoplasmic transport and destabilise the nuclear laminin leading to cellular damage. Interestingly, oligomeric tau induces astrocytic senescence and drives the release of HMGB1 which initiates further cellular senescence [39]. In our combined analysis of tau interactions, Musashi 2 and HMGB1 were found to interact with tau in one study—Tracy et al. [105]. These papers highlight the broad impact of tau on RNA binding proteins and potential to impact other cells in the brain if they can drive the release of HMGB1.

Only a small number of studies have focused on HNRNP interactions with tau. HNRNPA1 and HNRNPK co-localize with small tau inclusions but are not associated with large tau inclusions such as NFTs. A number of HNRNPs form their own separate insoluble aggregates as tau pathology progresses, such as HNRNPK in FTLD [14]. HNRNPK aggregates did not co-localize with large tau aggregates and were mutually exclusive of TDP43 pathology [14], but instead appeared in neighbouring neurons in vulnerable brain regions that did not contain tau inclusions. One possible explanation for this is that HNRNPs may interact with oligomeric tau species early in disease rather than fibrillar tau aggregates [79], a hypothesis explicitly supported by Jiang et al. [57]. HNRNPK modifies tau toxicity in *drosophila* models [6], suggesting that whilst HNRNPK aggregates do not appear to directly interact with tau in pathological inclusions, it is involved in mediating tau toxicity. Intracellular HNRNPK puncta were distinct from GS3BP2 stress granules, suggesting the aggregation of HNRNPK is separate from the TIA1-tau aggregation pathways, and TDP-43 positive inclusions [14]. Knock-down of HNRNPK in SH-SY5Y neuroblastoma cells resulted in similar splicing defects that are present in FTLD human brain tissue [14], suggesting that the accumulation of HNRNPs in tauopathy may result in loss of function. It is not yet known whether this loss of function is a result of stress granule formation or an earlier disruption of spliceosome function [54, 67, 83, 85].

HNRNPA2/B1 also mediates tau aggregation. Jiang et al. [57] showed that numerous HNRNPs interacted with tau oligomers, generated using an optogenetic approach in mouse primary neurons, including HNRNP A0, A1, A2B1, DL, F, H, K, L, M, R and UL1. Many ribosomal proteins also interacted with tau oligomers. Of these, HNRNPA2/B1 was the most significant tau oligomer interactor and interaction resulted in mislocalization of HNRNPA2/B1 from the nucleus to the cytoplasm *in vitro*, in mouse models of tauopathy and in human AD post-mortem brain tissue [57]. Importantly, reduction of HNRNPA2/B1 expression delayed tau oligomer formation and reduced toxicity. Curiously this interaction and tau oligomer toxicity was mediated by m^6^A RNA and knockdown of METTL3 (an m^6^A writer) delayed oligomer formation. This was correlated with increased prevalence of m^6^A labelled mRNA in AD with disease severity (fourfold in mouse models, 2.5 fold in AD brains) [57], suggesting that specific mRNA types are dysregulated with worsening severity in disease. Stable tau oligomer expression also elicits the aggregation of stress granule markers TIA1 and EIF3-η and PABP [57], but no enrichment for stress granule markers G3BP1, G3BP2, CAPRIN or USP10. This suggests there may be different subsets of stress granules that accumulate specific transcripts.This would cause tau:RNA binding protein interactions to sequester specific subsets of the mRNA pool and disproportionately impact the targeted pathways. Furthermore, HNRNPA2/B1 mediates the development of tau-containing stress granules and the interaction between tau oligomers and HNRNPA2/B1 alter protein synthesis [55]. Together, these results provide evidence for a concerted disruption of RNA metabolism by tau. Furthermore, the interactions between tau and RNA binding proteins may promote further dysregulation by over-activation of stress responses.

## Conclusions

The impacts of tau dysregulation through oligomerisation and aggregation in disease are likely conveyed by protein–protein (or protein-RNA) interactions specific to the brain region, sub-cellular compartment and cell type affected. As tau accumulates it can sequester interacting proteins and these interacting proteins can potentially modify the development of pathology. As tau pathology accumulates over the lifetime of a patient it has the potential to act as a time-capsule, preserving information about the early micro-environment that generated these aggregates. Our combined analysis of the human tau interactome with proteins present in neurons containing GVD or NFTs provides new information about the disease mechanisms that may be involved in tauopathies. Whilst many of the studies done to date have been limited in scope (i.e., have targeted a single tau isoform or a single disease), together these studies consistently identify many novel and overlooked protein interactions that may mediate toxicity in tauopathies. Our analysis has highlighted the particular importance of the interaction between tau and RNA binding proteins (particularly HNRNPs) in tauopathies. The consistent detection of these interactions across multiple human studies suggests that they are *bona fide* tau interactors.

There are still many unanswered questions in this field that require further research. For example, the vast majority of tau interactome studies performed to date have only focused on AD or models expressing mutated tau present in rare cases of FTLD. Similar experiments in other tauopathies or models using different tau isoforms would help to elucidate tau isoform and mutant specific interactions that may reveal novel disease mechanisms. Future mechanistic studies are required to determine whether specific protein interactions with tau influence pathology development and if these interactions could be therapeutically targeted. Initial studies suggest that disease associated PTMs change the interactome of tau, however, larger systematic studies are required to examine the impact of individual PTMs on the tau interactome across the spectrum of tauopathies.

A more detailed exploration of tau-protein interactions has the potential to reveal underlying disease mechanisms of tauopathies and to identify novel targets for drug development. Much more study is needed to accommodate the complexity of tau that results from the interaction of multiple tau isoforms, multiple different superstructures of aggregates and dysregulation in unique cells and regions in different diseases. It is critical we develop models that can fully accommodate this complexity and begin to move beyond single isoform/single disease approaches. Studies to date which investigate the mechanisms driving tau aggregation through interacting partners highlight the importance of understanding these interactions. Our combined analysis of tau interactome studies presented here highlight the potential importance of the interaction between tau and RNA binding proteins, particularly HNRNPs, in driving tauopathy. As such, exploring the mechanistic role of HNRNPs in tauopathy is an exciting future avenue of research.

## Supplementary Information

**Additional file 1: Supplementary Table 1.** List of post-translational modifications detected on tau across AD, AGD, CBD, PiD and PSP. **Supplementary Table 2.** Summary of human tau interactome and localised pathology proteomics studies. **Supplementary Table 3.** Summary of rodent Tau interactome studies. **Supplementary Table 4.** Gene Ontology Molecular Function Gene Sets and Enrichment Statistics. **Supplementary Table 5.** Summary of pathology targeted localised proteomics studies. **Supplementary Table 6.** GO cellular component comparison gene sets used for figure 3B.

## Data Availability

All data generated or analysed during this study are included in this published article (and its supplementary information files).

## Abbreviations

AD: Alzheimer’s disease
AGD: Argyrophilic grain disease
ARTAG: Aging related tau astrogliopathy
CBD: Corticobasal degeneration
CTE: Chronic traumatic encephalopathy
FTLD: Frontotemporal lobar degeneration
GGT: Globular glial tauopathy
GVD: Granulovacuolar degeneration
HNRNPs: Heterogeneous nuclear ribonucleoproteins
IPSCs: Induced pluripotent stem cells
NFTs: Neurofibrillary tangles
PART: Primary age related tauopathy
PiD: Pick’s disease
PSP: Progressive supranuclear palsy
PTM: Post-translational modification
pTau: Phosphorylated tau
RBP: RNA binding protein
